# New, Old and Forgotten Remedies

**Published:** 1918-02

**Authors:** 


**New, Old and Forgotten Remedies.** Papers by many writers. Second edition. Collected and arranged by Dr. Edward Pollock Anshutz, author of "Elements of Homoeopathic Theory, Materia Medica, Practice and Pharmacy," "Guide to the Twelve Tissue Remedies," etc. 608 pages. Cloth, $3.50 net. Philadelphia, Pa., Boericke & Tafel, 1917.

This is a book of which the founder of homoeopathy himself might have been proud. Truly, as the editor remarks, "Even some of Hahnemann's well proved remedies are practically unknown today. * * * The 'Test at the Bedside' * * * alone can determine the value of a new remedy." But this compilation shows that Hahnemann's soul is marching on even in our time, as is evidenced by the many provings of drugs "at the different homoeopathic colleges under modern conditions."

There are some 120 or more titles in the book, in alphabetic order, with a full "therapeutic and clinical index," listing many symptoms not commonly found in the literature of therapeutics, such as: anus burns, bad blood, dying babies, foul smelling body, pains beginning at head and going out at toes, itching in whiskers, and worse lying down. For each of these ills someone has named the appropriate cure, praise be to the "provings," but, unfortunately for the novice, the text does not always tell us which remedies are or should be "forgotten."

It is the province of the author to record the unusual and bizarre as well as the more conventional drugs of the materia medica. And so we have alfalfa, berberis, eucalyptus, ichthyol, etc., competing for recognition with such favorites as gunpowder, dog's milk, spider's venom, bacillinum, malaria officinalis, preparation of renal calculus, indol, and the saliva of the Gila monster (Heloderma horridus), all of which have had their provings and are still having their day. Many a foul remedy looks fair under its Latin designation, answering eloquently the poet's question "What's in a name?"

The author does not attempt to tell us how many authorities are quoted in this goodly volume. They are legion. He believes, however, that he has included in this edition "all the new remedies of value that have appeared in the past 17 years, together with those contained in the first edition (1900) now sold out." He hopes that the book "may tend to dispel in a measure the growing therapeutic nihilism,"

and so do we. The reviewer confesses that he is bothered considerably by this nihilism, and after reading the unvarnished and naive testimony of the "provers" quoted so frequently and so fully in this book, his hopes are considerably revived. Why would not a few doses of triturated cobwebs (Tela Araneæ) help to dispel the cobwebs in the editorial brain—although recommended specifically in 1876 for "an obstinate intermittent?"

The compiler has done his work conscientiously and faithfully. He does not speak for himself, however, except to advise the reader to "take the good and, in the current phrase of the hour, 'forget' the rest." This is all very well, but we imagine we detect a suspicion of humorous malice in his willingness to leave to the "kindly reader" the responsibility of taking and forgetting. We shall forget some of it, promptly, because the provers have not exhibited the discriminating knowledge of physiology and pathology which ought to be the common property of all medical practitioners. As for many other parts of the book we recognize that they represent an earnest search for more light on the elusive subject of therapeutics, and as such they are deserving of the study of every scientific physician.—F. W. B.

